# A parsimonious Langmuir-Freundlich loading-response framework for sonochemical degradation of nonvolatile organic contaminants: Model comparison, validation, and identifiability^☆^

**DOI:** 10.1016/j.ultsonch.2026.108033

**Published:** 2026-09-06

**Authors:** Oualid Hamdaoui

**Affiliations:** Chemical Engineering Department, College of Engineering, King Saud University, 12372 Riyadh, Saudi Arabia

**Keywords:** Sonochemical degradation, Langmuir–Freundlich kinetics, Model comparison, Internal validation, Parameter identifiability, Nonvolatile organic contaminants

## Abstract

A physically motivated Langmuir–Freundlich loading-response framework was formulated to describe the sonochemical degradation of nonvolatile organic contaminants in water. Acoustic cavitation was represented as a heterogeneous oxidative microenvironment in which contaminant degradation depends on apparent occupation of the bubble–liquid interfacial region and the finite oxidative capacity generated under fixed operating conditions. The framework was evaluated using naphthol blue black (NBB) and furosemide as chemically distinct nonvolatile contaminants. For NBB, increasing the initial concentration from 3 to 120 mg/L increased the initial degradation rate from 0.22268 to 2.1502 mg/L·min, while the 30-min removal efficiency decreased from 100 % to 43.46 % and the net H_2_O_2_ formation rate decreased from 5.4794 to 3.9161 μmol/L·min. Initial-rate calibration gave r_max_ = 3.8324 mg/L·min, K_LF_ = 1.1298 × 10^−2^ L/mg, and n = 0.8256. To reduce parameter redundancy, all concentration–time profiles for each compound were described using four global parameters, r_0,ref,T_, β_T_, K_LF,T_, and n_T_, without concentration-specific fitted coefficients. The calibration RMSE values were 0.0196 for NBB and 0.0399 for furosemide. Blocked leave-one-concentration-out validation gave pooled RMSE values of 0.0391 and 0.0566, respectively. For NBB, 30-min removal measurements at 7, 10, 40, and 120 mg/L, which were excluded from time-resolved parameter estimation, were calculated with an RMSE of 1.79 percentage points. Comparison with simpler formulations showed that the loading-dependent pseudo-first-order model gave the lowest cross-validated RMSE for NBB, whereas the classical Langmuir and Langmuir–Freundlich formulations gave comparable cross-validated performance for furosemide. Parameter confidence intervals and covariance-based correlation analysis identified strong compensation among the initial-rate parameters but lower correlations in the four-parameter time-resolved formulation. The fitted coefficients are therefore interpreted as condition-specific apparent descriptors rather than direct measurements of interfacial adsorption, radical flux, or molecular cooperativity. The framework provides a reduced-order tool for interpolation and treatment-time calculations within a calibrated operating domain, but application to other compounds, reactors, or solution matrices requires recalibration and independent validation.

## 1 Introduction

Organic micropollutants, synthetic dyes, and pharmaceutical residues are increasingly recognized as persistent contaminants in aquatic environments because many of these compounds are incompletely removed by conventional wastewater treatment processes [1], [2]. Their continuous release, structural stability, and potential ecological impacts have intensified interest in advanced oxidation processes capable of destroying contaminants rather than transferring them between phases [1], [2]. Among these technologies, high-frequency ultrasound has attracted sustained attention because acoustic cavitation can generate highly reactive oxidative microenvironments in water without the mandatory addition of external oxidants or catalysts [3], [4], [5], [6].

The chemical effects of ultrasound originate from the formation, growth, and implosive collapse of cavitation bubbles. Bubble collapse produces localized extreme conditions and promotes water sonolysis, generating reactive species such as hydroxyl radicals (HO^•^), hydrogen atoms (H^•^), as well as molecular products such as hydrogen peroxide (H_2_O_2_) [3], [4], [5], [6]. These species may recombine, diffuse into the liquid phase, or react with dissolved organic contaminants. Therefore, sonochemical degradation is governed by a dynamic competition between radical generation, radical recombination, contaminant oxidation, and transport of molecules toward cavitation-generated reactive zones [3], [4], [5].

For volatile contaminants, degradation may occur partly through pyrolysis inside the cavitation bubble because these compounds can partition into the gas phase during bubble growth. In contrast, nonvolatile organic contaminants are expected to degrade mainly at or near the bubble–liquid interface, and to a lesser extent in the surrounding bulk liquid, because their transfer into the cavitation bubble interior is negligible [5], [7], [8], [9], [10]. This distinction is critical for kinetic modeling: for nonvolatile molecules, the observed degradation rate is not controlled only by the bulk aqueous concentration, but also by the probability that contaminant molecules reach the short-lived HO^•^-rich interfacial reaction zone.

Many sonochemical degradation studies have interpreted concentration decay using pseudo-first-order kinetics because semilogarithmic concentration–time plots can appear approximately linear over restricted conversion intervals [5], [11]. However, this empirical simplification can obscure the heterogeneous nature of cavitation chemistry. Apparent rate constants are often affected by initial contaminant concentration, volatility, hydrophobicity, gas atmosphere, acoustic frequency, solution matrix, radical scavengers, and interfacial affinity [7], [8], [11], [12]. Consequently, a single pseudo-first-order coefficient cannot fully describe systems in which contaminant access to the cavitation interface and finite radical availability jointly control degradation.

A more mechanistic interpretation emerged from Langmuir-type sonochemical kinetic models. Serpone et al. [13] interpreted ultrasonic degradation of chlorophenols using a Langmuir-type treatment analogous to heterogeneous photocatalytic systems, suggesting that saturation-like behavior can arise when the reactive region becomes increasingly occupied by organic molecules. Kidak and Ince [14] later proposed an adsorption/saturation approach for ultrasonic phenol degradation, further supporting the role of local solute enrichment and finite radical availability. Okitsu et al. [8], [9] developed a heterogeneous kinetic model for azo dye degradation that explicitly considered the local concentrations of hydroxyl radicals and dyes near the cavitation bubble interface. These studies established the bubble–liquid interface as an effective reaction domain and provided the conceptual basis for interpreting nonvolatile contaminant sonodegradation as an interfacial heterogeneous process.

Subsequent work by Chiha et al. [15] systematically modeled ultrasonic degradation of nonvolatile organic compounds using Langmuir-type kinetics and showed that the initial degradation rate increases with contaminant concentration before approaching a limiting value. That work also emphasized the importance of nonlinear parameter estimation rather than relying only on linearized Langmuir transformations. More recently, Hamdaoui [16] derived general analytical solution expressions for Langmuir-type sonochemical degradation kinetics of nonvolatile organic contaminants in water, enabling rigorous analysis of full time-dependent concentration profiles rather than limiting the analysis to initial rate data. These contributions substantially advanced the mathematical treatment of Langmuir-type sonochemical kinetics; however, the classical Langmuir form still assumes an energetically uniform interfacial reaction domain.

The uniform interface assumption is restrictive for acoustic cavitation. The bubble–liquid boundary is transient, spatially nonuniform, and chemically heterogeneous. Local radical density, interfacial area, bubble curvature, collapse intensity, gas composition, temperature history, solute orientation, ionic strength, and contaminant speciation may vary continuously during ultrasonication. Therefore, contaminant occupation of the effective cavitation interface is unlikely to follow ideal single-site Langmuir behavior. A Langmuir–Freundlich, or Sips-type, formulation provides a more flexible structure because it retains the high-concentration saturation limit of the Langmuir model while introducing an exponent that accounts for nonideal interfacial occupation and distributed site affinities [17]. This flexibility makes the Langmuir–Freundlich expression a testable extension of the classical Langmuir formulation for sonochemical concentration–response data. However, the additional exponent does not independently establish interfacial heterogeneity and must be justified through parameter uncertainty, comparison with simpler nested alternatives, and out-of-sample performance.

In addition to contaminant degradation, H_2_O_2_ formation provides important information about radical utilization in sonochemical systems. H_2_O_2_ is formed mainly through radical recombination pathways; therefore, its formation rate can decrease when organic contaminants intercept hydroxyl radicals before recombination [3], [16]. Simultaneous analysis of contaminant degradation, net H_2_O_2_ formation, and treatment performance can therefore provide complementary evidence regarding changes in oxidant utilization as pollutant loading increases, although these macroscopic responses cannot by themselves establish a complete radical-partition balance.

In this work, a physically motivated Langmuir–Freundlich loading-response framework was examined for the sonochemical degradation of nonvolatile organic contaminants. Naphthol blue black, an azo dye, and furosemide, a pharmaceutical compound, were selected as structurally and physicochemically distinct substrates. The objectives were to: (i) determine whether a heterogeneous Langmuir–Freundlich expression provides information beyond classical Langmuir and loading-dependent pseudo-first-order formulations; (ii) describe complete concentration–time profiles using a continuous loading-dependent capacity function without assigning an independent coefficient to each initial concentration; (iii) distinguish calibration performance from out-of-sample validation; (iv) quantify parameter uncertainty, correlation, and practical identifiability; and (v) define the operating domain within which the formulation can be used for concentration-profile and treatment-time calculations. The fitted quantities are treated as apparent, condition-specific kinetic descriptors, and their mechanistic interpretation is restricted to consistency with an interfacial oxidation hypothesis rather than proof of a unique microscopic pathway.

## 2 Model development

The kinetic framework proposed in this study describes sonochemical degradation of nonvolatile organic contaminants as a heterogeneous interfacial oxidation process. The bulk aqueous phase is treated as the contaminant reservoir, whereas the cavitation bubble–liquid boundary is considered the dominant reactive region. This interpretation is consistent with previous Langmuir-type and heterogeneous interfacial descriptions of sonochemical degradation, in which nonvolatile contaminants react primarily at or near the cavitation interface rather than inside the bubble gas phase [8], [9], [15], [16]. The observed degradation rate is therefore controlled by two coupled factors: the fraction of contaminant molecules that effectively occupies the cavitation interfacial reaction zone and the finite oxidative capacity generated by acoustic cavitation under fixed operating conditions.

The fractional occupation of the effective interfacial oxidative capacity is described using a Langmuir–Freundlich, or Sips-type, expression, which extends the classical Langmuir saturation concept to heterogeneous interfacial domains [17]:

(1) $$\theta =\frac{{\left(K_{LF}\;C\right)}^{n}}{1+{\left(K_{LF}\;C\right)}^{n}}$$

where C is the bulk contaminant concentration, θ is an apparent bounded response function, K_LF_ is the Langmuir–Freundlich concentration-scale parameter, and n is the Langmuir–Freundlich curvature exponent. The product K_LF_C is dimensionless; therefore, K_LF_ has units of inverse concentration. When n = 1, Eq. (1) reduces to the classical Langmuir form. When n ≠ 1, the additional exponent allows systematic deviation from classical Langmuir curvature. Because K_LF_ and n are inferred from bulk concentration data, neither should be interpreted as a direct measurement of adsorption affinity, interfacial coverage, site-energy distribution, or molecular cooperativity.

The degradation rate is represented as proportional to the apparent response function:

(2) $$r=-\frac{dC}{dt}=r_{max}\theta$$

where r_max_ is the maximum observable sonochemical degradation rate under the applied ultrasonic conditions. Substituting Eq. (1) into Eq. (2) gives the proposed Langmuir–Freundlich interfacial degradation model:

(3) $$-\frac{dC}{dt}=r_{max}\frac{{\left(K_{LF}\;C\right)}^{n}}{1+{\left(K_{LF}\;C\right)}^{n}}$$

Eq. (3) is the principal kinetic expression of the framework. The parameter r_max_ is the limiting apparent degradation-rate scale under the applied reactor and acoustic conditions. The parameter K_LF_ defines the concentration scale over which the occupation function changes from its low-concentration regime toward saturation, whereas n controls the curvature of this transition. These quantities are inferred from bulk concentration data and should not be interpreted as direct measurements of interfacial surface coverage, adsorption energy, radical flux, or site heterogeneity. In a transient cavitation field, they may incorporate coupled effects of contaminant transport, speciation, interfacial enrichment, local radical availability, and spatially and temporally variable bubble dynamics.

The limiting apparent degradation-rate scale may be conceptually decomposed as:

(4) $$r_{max}={\frac{A_{eff}}{V}k}_{ox}\Gamma_{max}$$

where k_ox_ is the pseudo-oxidation rate constant for the interfacially accumulated contaminant, Γ_max_ is the maximum interfacial contaminant loading per unit area, A_eff_ is the effective cavitation interfacial area available for reaction, and V is the solution volume. If the oxidation step is explicitly related to the local hydroxyl radical flux, then:

(5) $$k_{ox}=k_{{HO}^{\cdot }}J_{{HO}^{\cdot }}$$

and consequently:

(6) $$r_{max}={\frac{A_{eff}}{V}k}_{{HO}^{\cdot }}J_{{HO}^{\cdot }}\Gamma_{max}$$

where $J_{{HO}^{\cdot }}$ is the local interfacial hydroxyl radical flux and $k_{{HO}^{\cdot }}$ is an efficiency coefficient linking radical flux to contaminant oxidation. This formulation separates the chemical oxidative strength of the cavitation field from the physicochemical tendency of the contaminant to access the reactive interface.

Eqs. (4), (5), (6) provide a conceptual decomposition of the apparent rate scale. The individual quantities A_eff_, k_HO•_, J _HO•_, and Γ_max_ cannot be identified separately from the bulk concentration–time measurements used in this study. Consequently, the fitted value of r_max_ represents their combined apparent contribution and does not independently establish the magnitude of any individual interfacial or radical-flux term.

The limiting forms of Eq. (3) provide direct kinetic interpretation. At low contaminant concentration, where (K_LF_ C)^n^ ≪ 1, Eq. (3) becomes:

(7) $$-\frac{dC}{dt}\approx r_{max}{\left(K_{LF}\;C\right)}^{n}$$

Thus, the apparent low-concentration kinetic order is n, rather than unity. This limiting behavior describes the curvature of the concentration response and does not by itself identify its microscopic origin. At high contaminant concentration, where (K_LF_ C)^n^ ≫ 1, Eq. (3) approaches:

(8) $$-\frac{dC}{dt}\approx r_{max}$$

which corresponds mathematically to the zero-order saturation limit of the Langmuir–Freundlich response. The apparent half-response concentration is obtained when θ = 0.5:

(9) $$K_{LF}C_{1/2}=1$$

(10) $$C_{1/2}=\frac{1}{K_{LF}}$$

Accordingly, larger K_LF_ values correspond mathematically to lower apparent half-response concentrations. Because K_LF_ is estimated from bulk kinetic data, this relationship should not be interpreted as a direct measurement of interfacial enrichment or adsorption affinity.

For initial degradation rate analysis, Eq. (3) is evaluated at C = C_0_:

(11) $$r_{0}=r_{max}\frac{{\left(K_{LF}\;C_{0}\right)}^{n}}{1+{\left(K_{LF}\;C_{0}\right)}^{n}}$$

where r_0_ is the initial degradation rate and C_0_ is the initial contaminant concentration. Nonlinear regression of r_0_ versus C_0_ enables estimation of r_max_, K_LF_, and n. In principle, the high-concentration behavior primarily constrains r_max_, the transition region informs K_LF_, and the low-concentration curvature provides information on n.

For batch degradation profiles, integration of Eq. (3) gives the concentration–time relationship. Rearranging Eq. (3):

(12) $$dt=-\frac{1}{r_{max}}\left[1+{\left(K_{LF}\;C\right)}^{-n}\right]dC$$

For n ≠ 1, integration from C_0_ at t = 0 to C at time t gives:

(13) $$t=\frac{C_{0}-C}{r_{max}}+\frac{C_{0}^{1-n}-C^{1-n}}{r_{max}K_{LF}^{n}\left(1-n\right)}$$

For the special case n = 1, Eq. (3) reduces to:

(14) $$-\frac{dC}{dt}=r_{max}\frac{K_{LF}\;C}{1+K_{LF}\;C}$$

and the corresponding integrated form becomes:

(15) $$t=\frac{C_{0}-C}{r_{max}}+\frac{1}{r_{max}K_{LF}}ln\left(\frac{C_{0}}{C}\right)$$

Thus, the proposed Langmuir–Freundlich model preserves the classical Langmuir-type sonochemical expression as a limiting case while introducing an additional exponent to describe interfacial nonideality. For n = 1, the explicit concentration–time solution can also be written using the Lambert W function, consistent with analytical treatments of Langmuir-type sonochemical kinetics [16]:

(16) $$C\left(t\right)=\frac{1}{K_{LF}}W\left[K_{LF}C_{0}e^{\left(K_{LF}C_{0}-K_{LF}r_{max}t\right)}\right]$$

where W is the Lambert W function, defined by W(x)e^W(x)^ = x.

The present study deliberately restricts the fitted framework to the minimum set of quantities identifiable from the available concentration–time data. Effects of contaminant speciation, solution-matrix constituents, radical scavengers, and explicit adsorption/desorption dynamics could in principle be incorporated by allowing the apparent kinetic parameters to depend on these variables. Such extensions would introduce additional coefficients that cannot be identified from the present dataset and are therefore not included in the fitted formulation. This restriction avoids parameter proliferation and focuses the subsequent analysis on observable concentration–response behavior, parameter uncertainty, and out-of-sample performance.

## 3 Materials and methods

### 3.1 Chemicals and solutions

All aqueous solutions were prepared using Milli-Q ultrapure water. Analytical grade reagents were used without further purification. Naphthol blue black (NBB), furosemide, and ammonium heptamolybdate were obtained from Sigma-Aldrich, whereas potassium iodide and acetonitrile were supplied by Riedel-de Haën. Stock solutions of NBB and furosemide were prepared in ultrapure water and diluted to the desired initial concentrations immediately before sonochemical experiments. The working solution volume was fixed at 300 mL for all degradation and hydrogen peroxide formation experiments.

The initial concentration ranges were selected separately for the two contaminants because of their markedly different aqueous solubilities and were therefore not intended to represent matched mass-concentration domains. NBB is substantially more soluble in water and could be investigated over the broader range of 3–120 mg/L. In contrast, the lower aqueous solubility of furosemide restricted its experimentally accessible concentration range; accordingly, furosemide solutions were investigated between 0.5 and 20 mg/L to maintain homogeneous dissolved-phase conditions and avoid introducing solubility-related limitations into the kinetic analysis. Within these compound-specific solubility constraints, the concentration windows were selected to provide sufficiently broad variation in pollutant loading to resolve the corresponding concentration-dependent kinetic behavior. For NBB, complete concentration–time profiles were obtained at 3, 5, 15, 20, 30, and 80 mg/L, while additional 30-min measurements at 7, 10, 40, and 120 mg/L extended the concentration–response assessment. For furosemide, complete concentration–time profiles were obtained over 0.5–20 mg/L.

### 3.2 Sonoreactor and ultrasonication procedure

The experimental concentration–time datasets analyzed in this study were generated in our previous investigations of NBB and furosemide sonodegradation and are reanalyzed here for the distinct purpose of developing, comparing, and validating the present loading-response kinetic framework [[18], [19]]. The present analysis, including the global parameterization, blocked leave-one-concentration-out validation, held-out concentration-endpoint assessment, model comparison, and identifiability analysis, has not been reported previously.

Ultrasonication experiments were carried out using a Meinhardt Ultrasonics high-frequency reactor, model E/805/T/M. The reactor consisted of a jacketed glass vessel containing 300 mL of aqueous solution. A cooling fluid was circulated continuously through the external jacket to maintain the reaction temperature at 25 ± 1 °C during irradiation. Ultrasonic waves were generated by a bottom-mounted transducer with an active diameter of 5.3 cm, operating at 585 kHz. The acoustic intensity was set to 3.58 W/cm^2^ for NBB degradation and 4.3 W/cm^2^ for furosemide degradation. The effective ultrasonic power delivered to the liquid was determined by calorimetry [20].

During ultrasonication, aliquots were withdrawn from the reactor at predetermined irradiation times for analysis. For NBB experiments, samples were analyzed spectrophotometrically. For furosemide experiments, samples were analyzed by HPLC. Hydrogen peroxide formation was quantified independently using the iodometric method. Dye-free experiments were also carried out to determine the baseline sonochemical formation rate of hydrogen peroxide in ultrapure water.

Control experiments were conducted in the absence of ultrasonic irradiation to assess possible pollutant losses unrelated to sonochemical activity. NBB and furosemide solutions were placed in the same reactor with the ultrasonic transducer switched off and were subjected only to stirring at 400 rpm while the temperature was maintained at 25 °C. The solution volume, sampling procedure, treatment duration, and analytical methods were otherwise identical to those employed in the corresponding ultrasonic degradation experiments. These ultrasound-off controls were used to assess the stability of the contaminants and to determine whether mechanical mixing, spontaneous transformation, or interaction with the experimental system contributed measurably to pollutant removal.

### 3.3 Analytical methods

The concentration of NBB was determined using a UV–visible Lightwave II spectrophotometer (Biochrom WPA). Absorbance was measured at 620 nm, corresponding to the characteristic absorption wavelength of NBB. Concentrations were calculated from calibration curves prepared under the same analytical conditions.

Furosemide was quantified using a YL Instruments HPLC system (YL9100) equipped with a YL9120 UV–visible detector. Separation was performed on a Supelcosil LC-18 column (250 × 4.6 mm, 4 μm). The detector wavelength was set to 230 nm. The mobile phase consisted of water:acetonitrile (70:30, v/v) containing 0.1 % formic acid, operated under isocratic elution at a flow rate of 0.6 mL/min. Furosemide concentrations were determined from calibration curves prepared using standard solutions.

The concentration of sonochemically generated hydrogen peroxide was determined by iodometry [21]. For each measurement, 200 μL of sample withdrawn from the reactor was mixed in a quartz cuvette with 1000 μL of 0.1 M potassium iodide and 20 μL of 0.01 M ammonium heptamolybdate. After 5 min of reaction time, the absorbance was measured at 350 nm using the UV–visible spectrophotometer. Hydrogen peroxide formation rates were calculated from the initial linear accumulation region.

### 3.4 Parameter estimation, uncertainty, model comparison, and validation

All kinetic parameters were estimated by nonlinear regression without linearization of the governing equations. The initial-rate parameters r_max_, K_LF_, and n were obtained by fitting Eq. (11) to the measured initial NBB degradation rates. The H_2_O_2_-response parameter $\Delta r_{H_{2}O_{2}}$ was estimated using the occupation function obtained from the initial-rate analysis. The quantity Ψ = $\Delta r_{H_{2}O_{2}}$/r_max_ was calculated from the fitted parameters and was not treated as an independently adjustable coefficient. Because the numerator and denominator have different concentration units, Ψ is an empirical coupling slope with units of μmol/mg, rather than a dimensionless radical-partition fraction.

Removal efficiency was not fitted using a separate empirical performance equation. At any treatment time t, it was calculated directly from the concentration predicted by the time-resolved formulation:

(17) $$E\left(t,\;C_{0}\right)=100\left[1-\frac{C\left(t,\;C_{0}\right)}{C_{0}}\right]$$

The NBB removal values shown in Fig. 2 correspond to an ultrasonication time of 30 min. Complete concentration–time profiles at 3, 5, 15, 20, 30, and 80 mg/L were used for time-resolved calibration. The 30-min removal measurements at 7, 10, 40, and 120 mg/L were excluded from time-resolved parameter estimation and were reserved as a held-out concentration-endpoint assessment.

For the time-resolved analysis, the apparent oxidative capacity was represented as a continuous function of initial pollutant loading. A fixed reference concentration of C_ref_ = 10 mg/L was used:

(18) $$R_{T}\left(C_{0}\right)=\frac{r_{0,ref,T}}{\theta_{ref}}{\left(\frac{C_{0}}{C_{ref}}\right)}^{-\beta_{T}}$$

where

(19) $$\theta_{ref}=\frac{{\left(K_{LF,T}\;C_{ref}\right)}^{n_{T}}}{1+{\left(K_{LF,T}\;C_{ref}\right)}^{n_{T}}}$$

The parameter r_0,ref,T_ is the calculated initial degradation rate at C = C_0_ = C_ref_, and β_T_ describes the systematic variation in apparent oxidative capacity with initial loading. All concentration–time profiles for a given compound were fitted simultaneously using four shared parameters: r_0,ref,T_, β_T_, K_LF,T_, and n_T_. No parameter was fitted independently for an individual concentration profile.

The corresponding time-resolved rate equation is:

(20) $$-\frac{dC}{dt}=R_{T}\left(C_{0}\right)\frac{{\left(K_{LF,T}\;C\right)}^{n_{T}}}{1+{\left(K_{LF,T}\;C\right)}^{n_{T}}}$$

For n_T_ ≠ 1, integration from C_0_ at t = 0 to C_t_ at time t gives:

(21) $$t=\frac{C_{0}-C_{t}}{R_{T}\left(C_{0}\right)}+\frac{C_{0}^{1-n_{T}}-C_{t}^{1-n_{T}}}{R_{T}\left(C_{0}\right)K_{LF,T}^{n_{T}}\left(1-n_{T}\right)}$$

Equations (18), (19), (20), (21) constitute the four-parameter time-resolved formulation used for both NBB and furosemide. When n_T_ = 1, Eq. (20) reduces to the nested classical Langmuir form. The parameter architecture is modular rather than a single high-dimensional regression. For NBB, the complete analysis reports eight fitted quantities distributed among three separate response modules: three initial-rate parameters (r_max_, K_LF_, and n), one H_2_O_2_-response amplitude ($\Delta r_{H_{2}O_{2}}$), and four time-resolved parameters (r_0,ref,T_, β_T_, K_LF,T_, and n_T_). These quantities are not estimated simultaneously. Only the four time-resolved parameters are required to calculate C(t), removal efficiency, or the treatment time required to reach a prescribed residual concentration. For furosemide, only the four time-resolved parameters are estimated in the present analysis. Removal efficiency introduces no fitted parameter. Thus, the concentration-specific coefficients responsible for parameter proliferation are eliminated from the predictive formulation.

Parameter uncertainty was evaluated from the nonlinear-regression covariance matrix. Standard errors, relative standard errors, and approximate 95 % Wald confidence intervals were calculated for each fitted parameter. Confidence intervals for reciprocal quantities, including C_1/2_ = 1/K_LF_, were obtained by transforming the confidence limits of the corresponding concentration-scale parameter. Pairwise parameter-correlation coefficients were calculated as:

(22) $$\rho_{ij}=\frac{cov(p_{i},p_{j})}{\sqrt{var\left(p_{i}\right)var\left(p_{j}\right)}}$$

Absolute correlation coefficients approaching unity indicate strong parameter compensation and reduced practical identifiability. The covariance-based standard errors, confidence intervals, and correlations characterize local practical identifiability in the neighborhood of the nonlinear-regression optimum. They do not constitute a formal proof of global structural identifiability.

Internal out-of-sample transfer across pollutant loading was evaluated using blocked leave-one-concentration-out validation. In each fold, one complete concentration–time profile was withheld, all model parameters were estimated using the remaining profiles, and the excluded trajectory was calculated without parameter re-estimation. This procedure was repeated until every initial concentration had served as the withheld profile. The pooled validation RMSE was calculated from all withheld observations.

The Langmuir–Freundlich formulation was compared with two simpler alternatives using the same concentration–time observations and validation folds. The first alternative was a loading-dependent pseudo-first-order model:

(23) $$-\frac{dC}{dt}=k_{ref}{\left(\frac{C_{0}}{C_{ref}}\right)}^{-\gamma }C$$

where k_ref_ is the apparent first-order coefficient at C_ref_, and γ describes its loading dependence. The second alternative was the classical Langmuir formulation obtained by constraining n_T_ = 1. The pseudo-first-order, classical Langmuir, and Langmuir–Freundlich formulations contained two, three, and four globally estimated parameters, respectively. Their performances were compared using calibration RMSE and pooled blocked leave-one-concentration-out RMSE.

Statistics calculated from observations used for parameter estimation are reported explicitly as calibration statistics. Blocked leave-one-concentration-out results are described as internal out-of-sample validation because the withheld profiles were obtained using the same reactor and operating conditions as the calibration profiles. The NBB measurements at 7, 10, 40, and 120 mg/L constitute a held-out concentration-endpoint assessment because they were not used to estimate the time-resolved parameters. Neither assessment establishes external transfer to another sonoreactor, acoustic condition, solution matrix, laboratory, or independently collected furosemide dataset.

## 4 Results and discussion

### 4.1 Concentration dependence and apparent saturation behavior of NBB sonodegradation

Control experiments conducted in the absence of ultrasonic irradiation showed that the concentrations of both NBB and furosemide remained essentially unchanged over the investigated periods. Thus, pollutant losses under stirring-only conditions were negligible compared with the concentration decreases observed during ultrasonic irradiation. These results indicate that mechanical mixing, spontaneous transformation, and interaction with the experimental system did not contribute appreciably to pollutant removal. The degradation observed during ultrasonic irradiation can therefore be attributed predominantly to ultrasound-induced sonochemical processes under the investigated conditions.

**Fig. 1:**
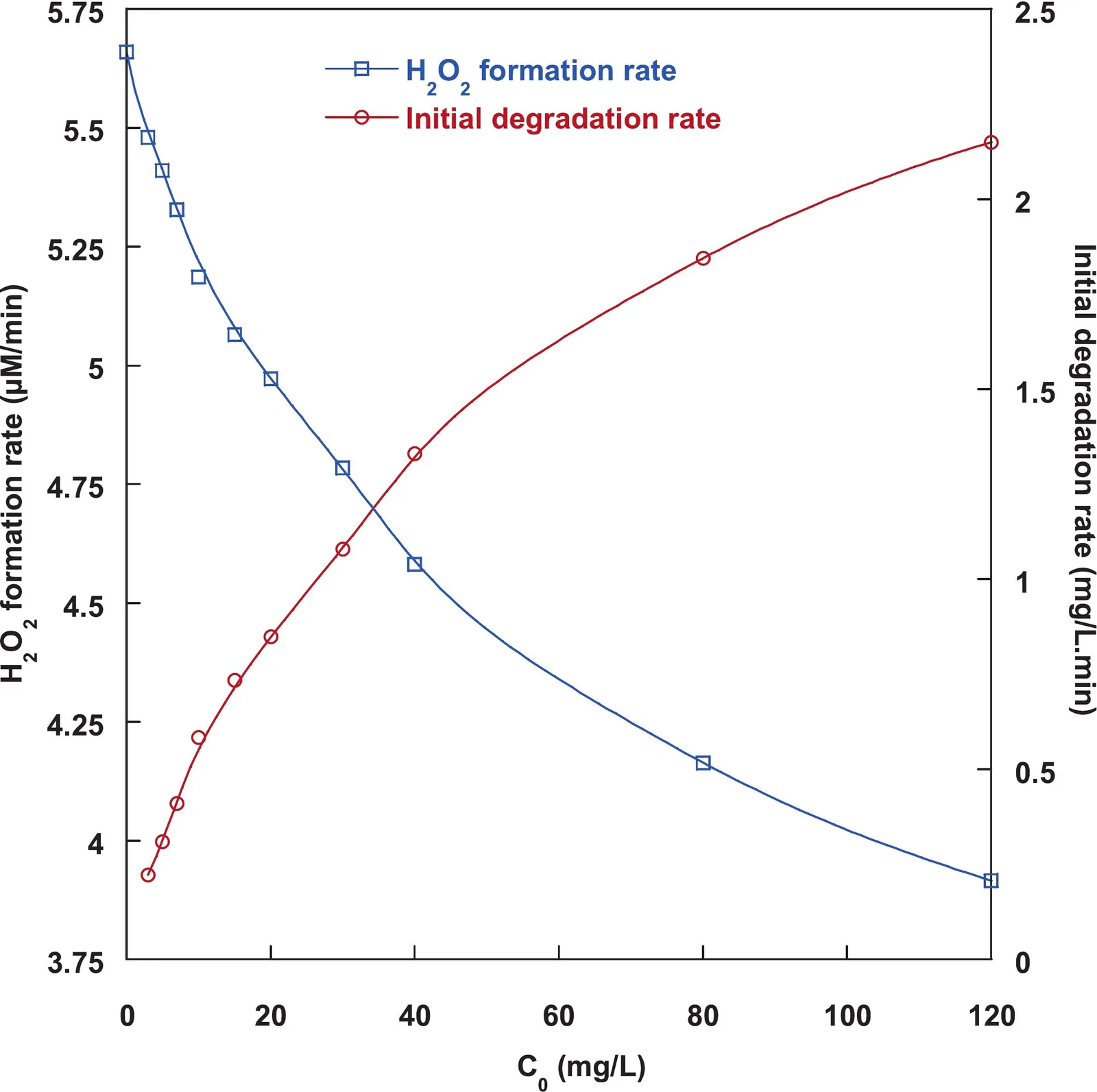
Experimental H_2_O_2_ formation rates and initial NBB degradation rates as functions of initial concentration. Symbols represent experimental observations, and solid lines represent calibration fits obtained using the corresponding models. The fitted curves should not be interpreted as independent predictions.

**Fig. 2:**
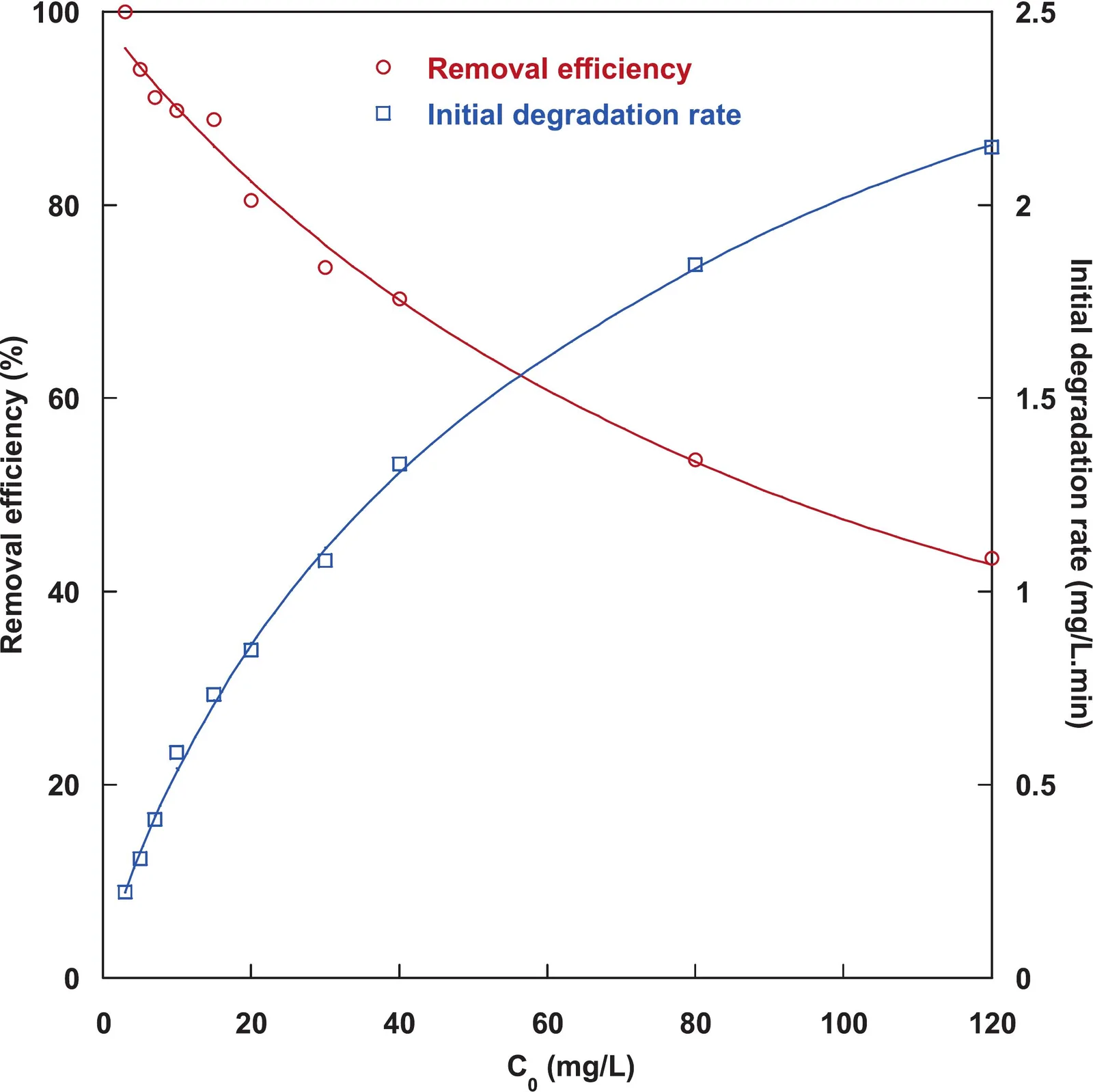
Experimental and model-calculated 30-min NBB removal efficiencies and model-fitted initial degradation rates as functions of initial concentration. The removal-efficiency curve was calculated from the time-resolved concentration model without fitting an additional performance parameter. The removal measurements at 7, 10, 40, and 120 mg/L were not used to estimate the time-resolved parameters and provide a held-out concentration-endpoint assessment.

Fig. 1, Fig. 2 summarize the concentration-dependent NBB responses observed during sonochemical treatment. Increasing the initial concentration, C_0_, from 3 to 120 mg/L increased the initial degradation rate, r_0_, from 0.22268 to 2.1502 mg/L·min. In contrast, the 30-min removal efficiency decreased from 100 % to 43.46 %, while the net H_2_O_2_ formation rate decreased from 5.4794 to 3.9161 μM/min, compared with 5.66 μM/min in dye-free water.

These opposing trends are compatible with a finite-capacity concentration response and increased competition between organic oxidation and pathways contributing to net H_2_O_2_ accumulation. However, they do not uniquely identify interfacial occupation, radical transport, or radical-partition mechanisms. The observed response may incorporate contaminant transport, speciation, interfacial enrichment, radical interception, intermediate reactions, and variations in the transient cavitation field [8], [9], [15], [16]. Within the present framework, the sublinear increase in r0 is represented by a bounded apparent occupation function, but this function should not be interpreted as a direct measurement of molecular surface coverage.

The initial degradation rate data were fitted using the Langmuir–Freundlich interfacial expression:

(24) $$r_{0}\left(C_{0}\right)=r_{max}\frac{{\left(K_{LF}\;C_{0}\right)}^{n}}{1+{\left(K_{LF}\;C_{0}\right)}^{n}}$$

where r_max_ is the limiting apparent initial degradation-rate scale, K_LF_ is the Langmuir–Freundlich concentration-scale parameter, and n is the Langmuir–Freundlich curvature exponent.

Nonlinear regression gave r_max_ = 3.8324 ± 0.3401 mg/L·min, K_LF_ = (1.1298 ± 0.2425) × 10^−2^ L/mg, and n = 0.8256 ± 0.0395, where the uncertainties are standard errors. The corresponding relative standard errors were 8.87 %, 21.47 %, and 4.79 %, respectively, and the approximate 95 % confidence intervals were 3.1659–4.4989 mg/L·min, 6.545 × 10^−3^–1.6051 × 10^−2^ L/mg, and 0.7481–0.9031. The calibration RMSE was 0.0208 mg/L·min. Because the same observations were used for parameter estimation and evaluation of this residual error, the value describes calibration performance and not independent predictive performance.

The initial-rate parameters were strongly correlated. The maximum absolute pairwise coefficient was 0.996, occurring between r_max_ and K_LF_, while the K_LF_–n and r_max_–n correlations were 0.960 and − 0.944, respectively. The parameters should therefore be interpreted jointly. In particular, the available data constrain the observed rate curve more strongly than they constrain a unique decomposition into limiting capacity, concentration scale, and curvature.

The fitted value of r_max_ represents the limiting apparent initial-rate scale under the applied sonochemical conditions. As discussed in Section 2, this quantity may incorporate several coupled physical and chemical contributions that cannot be identified separately from the bulk kinetic data. Similarly, K_LF_ defines the apparent concentration scale over which the fitted response changes curvature and should not be interpreted as an independently measured adsorption or interfacial-affinity constant. The corresponding apparent half-response concentration is:

(25) $$C_{1/2}=\frac{1}{K_{LF}}=88.5\;mg/L$$

Transformation of the confidence limits of K_LF_ gave an approximate interval of 62.3–152.8 mg/L. Because this interval extends beyond the upper part of the investigated concentration range, the saturation plateau was not fully constrained by the initial-rate observations. This limitation is consistent with the strong correlation between r_max_ and K_LF_. Accordingly, C_1/2_ is interpreted as an uncertain apparent concentration scale rather than a directly measured interfacial saturation concentration.

The fitted Langmuir–Freundlich exponent was lower than unity (n = 0.8256), indicating measurable curvature relative to the classical Langmuir case n = 1. This deviation may reflect several coupled effects, including contaminant transport, speciation, interfacial enrichment, local oxidant availability, and the transient nature of the cavitation field. Because these contributions were not measured independently, n is interpreted as an apparent kinetic curvature parameter rather than as a direct measure of surface heterogeneity or a particular distribution of interfacial site energies.

The predicted fractional occupation of the effective oxidative interface can be estimated from:

(26) $$\theta \left(C_{0}\right)=\frac{r_{0}{(C}_{0})}{r_{max}}$$

Using the fitted parameters, the apparent occupation function increased from approximately 0.058 at 3 mg/L to 0.563 at 120 mg/L. These values are model-derived dimensionless quantities and not experimentally measured interfacial coverages. They indicate that the calibrated response remained within the transition region of the mathematical saturation function over the investigated concentration range.

At low concentration, where (K_LF_C_0_)^n^ ≪ 1, Eq. (24) reduces to:

(27) $$r_{0}\left(C_{0}\right)\approx r_{max}K_{LF}^{n}C_{0}^{n}$$

Using the fitted parameters:

(28) $$r_{0}\left(C_{0}\right)\approx 0.0946C_{0}^{0.8256}$$

This fractional-order relationship shows that the calibrated initial degradation rate increases sublinearly with C_0_. For example, doubling C_0_ increases the fitted rate by a factor of 2^0.8256^ = 1.77, rather than by a factor of 2. This behavior is consistent with a heterogeneous or transport-influenced concentration response but does not uniquely establish its microscopic origin.

The limitation of a concentration-independent pseudo-first-order interpretation is further illustrated by the apparent rate coefficient:

(29) $$k_{app}=\frac{r_{0}}{C_{0}}$$

The experimental value of k_app_ decreased from 0.0742 min^−1^ at 3 mg/L to 0.0179 min^−1^ at 120 mg/L. This approximately fourfold decrease shows that the apparent kinetic coefficient is not constant, but depends strongly on the initial dye loading. Therefore, the observed degradation cannot be described as a concentration-independent pseudo-first-order process over the investigated concentration range. This result excludes the use of a single concentration-independent pseudo-first-order coefficient across the complete loading range. It does not exclude a loading-dependent pseudo-first-order formulation, which is evaluated explicitly against the classical Langmuir and Langmuir–Freundlich models in Section 4.6.

The initial-rate data are therefore compatible with a finite-capacity, sublinear concentration response. The Langmuir–Freundlich expression provides a compact and physically motivated representation of this response, but its calibration quality does not prove a unique interfacial mechanism. The fitted coefficients may combine the effects of pollutant transport, speciation, interfacial enrichment, transient radical availability, and the dynamic cavitation field. Their principal value is to summarize the observed concentration dependence and to provide testable concentration-scale and curvature descriptors for comparison with simpler formulations.

### 4.2 Empirical association between NBB degradation and net H_2_O_2_ formation

The simultaneous increase in the initial NBB degradation rate and decrease in the net H_2_O_2_ formation rate is compatible with competition between organic oxidation and pathways contributing to H_2_O_2_ accumulation. In dye-free water, hydroxyl-radical recombination contributes to H_2_O_2_ formation. In the presence of NBB, reactions involving the dye and its transformation products may decrease the amount of H_2_O_2_ accumulated in the liquid. However, the measured H_2_O_2_ formation rate is a net response that may include both H_2_O_2_ production and consumption. It therefore cannot be used alone to determine the fraction of hydroxyl radicals reacting with NBB or to establish a complete radical balance.

To represent the observed concentration dependence without introducing a separate occupation function, the H_2_O_2_ formation rate was expressed using the apparent Langmuir–Freundlich function obtained from the initial-rate analysis:

(30) $$r_{H_{2}O_{2}}\left(C_{0}\right)=r_{H_{2}O_{2},0}-\Delta r_{H_{2}O_{2}}\theta \left(C_{0}\right)$$

where $r_{H_{2}O_{2}}\left(C_{0}\right)$ is the measured net H_2_O_2_ formation rate at a given initial NBB concentration, $r_{H_{2}O_{2},0}$ is the corresponding rate in dye-free water, and $\Delta r_{H_{2}O_{2}}$ is the fitted limiting amplitude of the decrease associated with the apparent occupation function. The parameter is an empirical response amplitude and not a direct measure of radical diversion.

Using $r_{H_{2}O_{2},0}$ = 5.66 μM/min, $\Delta r_{H_{2}O_{2}}$ = 3.1082 μM/min, and the initial-rate parameters, the numerical expression was:

(31) $$r_{H_{2}O_{2}}\left(C_{0}\right)=5.66-3.1082\frac{{\left(1.1298\times 10^{-2}\;C_{0}\right)}^{0.8256}}{1+{\left(1.1298\times 10^{-2}\;C_{0}\right)}^{0.8256}}$$

Nonlinear regression gave $\Delta r_{H_{2}O_{2}}$ = 3.1082 ± 0.0193 μM/min, corresponding to a relative standard error of 0.62 % and an approximate 95 % confidence interval of 3.0703–3.1461 μM/min. The calibration RMSE was 0.0168 μM/min. Because $\Delta r_{H_{2}O_{2}}$ was estimated from these same observations, this value quantifies calibration error rather than independent predictive performance.

Because the modeled initial degradation rate and H_2_O_2_ response share the same apparent occupation function, eliminating θ(C_0_) gives:

(32) $$r_{H_{2}O_{2}}\left(C_{0}\right)=r_{H_{2}O_{2},0}-\Psi {\;r}_{0}\left(C_{0}\right)$$

where Ψ is an empirical coupling slope between the net H_2_O_2_ formation rate and the initial NBB degradation rate. Because the two rates are expressed using different concentration units, Ψ has units of μmol/mg and is not a dimensionless fraction of the radical flux:

(33) $$\Psi =\frac{\Delta r_{H_{2}O_{2}}}{r_{max}}=\frac{3.1082}{3.8324}=0.8110\;\mu mol/mg$$

Propagation of the fitted-parameter uncertainties gave Ψ = 0.8110 ± 0.0721 μmol/mg, with an approximate 95 % confidence interval of 0.670–0.953 μmol/mg. The corresponding empirical relationship was:

(34) $$r_{H_{2}O_{2}}\left(C_{0}\right)=5.66-0.8110\;r_{0}\left(C_{0}\right)$$

Eq. (34) is an algebraic consequence of the shared concentration–response function and the fitted H_2_O_2_ amplitude. It represents the observed inverse association under the applied conditions but does not constitute independent validation of a radical-partition mechanism. In particular, it does not account explicitly for radical stoichiometry, H_2_O_2_ consumption, intermediate oxidation, or competing radical pathways.

Removal efficiency was calculated directly using Eq. (17) and therefore introduced no additional fitted parameter.

### 4.3 Parsimonious time-resolved modeling and loading-based validation of NBB degradation

The NBB concentration–time profiles were analyzed using the four-parameter formulation defined by Eqs. (18), (19), (20), (21). This representation replaces concentration-specific capacity coefficients with the continuous loading-response function R_T_(C_0_), thereby permitting calculation at initial concentrations not explicitly represented by a fitted local coefficient. All 83 NBB observations were fitted simultaneously using the shared parameters r_0,ref,T_, β_T_, K_LF,T_, and n_T_.

Nonlinear regression gave r_0,ref,T_ = 0.7284 ± 0.0156 mg/L·min, β_T_ = 0.3354 ± 0.0249, K_LF,T_ = (5.322 ± 1.586) × 10^−3^ L/mg, and n_T_ = 0.9422 ± 0.0265. The resulting loading-dependent apparent capacity was:

(35) $$R_{T}\left(C_{0}\right)=12.2815{\left(\frac{C_{0}}{10}\right)}^{-0.3354}\;mg/L\cdot min$$

and the complete numerical rate equation for NBB was:

(36) $$-\frac{dC}{dt}=12.2815{\left(\frac{C_{0}}{10}\right)}^{-0.3354}\frac{{\left(0.005322\;C\right)}^{0.9422}}{1+{\left(0.005322\;C\right)}^{0.9422}}$$

The global calibration RMSE was 0.0196 in normalized concentration units. Parameter estimates and uncertainty are summarized in Table 1. Relative standard errors ranged from 2.15 % to 29.80 %, with K_LF,T_ being the least precisely estimated parameter. The pairwise parameter correlation structure is reported in Table 4.

**Table 1:** Global time-resolved parameter estimates and uncertainty for NBB degradation.

**Parameter or statistic**	**Value**	**RSE (%)**	**Approximate 95 % CI**
r_0,ref,T_ (mg/L·min)	0.7284 ± 0.0156	2.15	0.6977–0.7590
β_T_	0.3354 ± 0.0249	7.43	0.2865–0.3842
K_LF,T_ (L/mg)	(5.322 ± 1.586) × 10^−3^	29.80	(2.214–8.430) × 10^−3^
n_T_	0.9422 ± 0.0265	2.82	0.8902–0.9942
C_1/2,T_ (mg/L), calculated	187.91	−	118.63–451.74

Note: All concentration–time profiles were fitted simultaneously using four common parameters and C_ref_ = 10 mg/L. Values following ± are standard errors obtained from nonlinear regression. C_1/2,T_ = 1/K_LF,T_ is a calculated quantity and was not independently fitted.

**Fig. 3:**
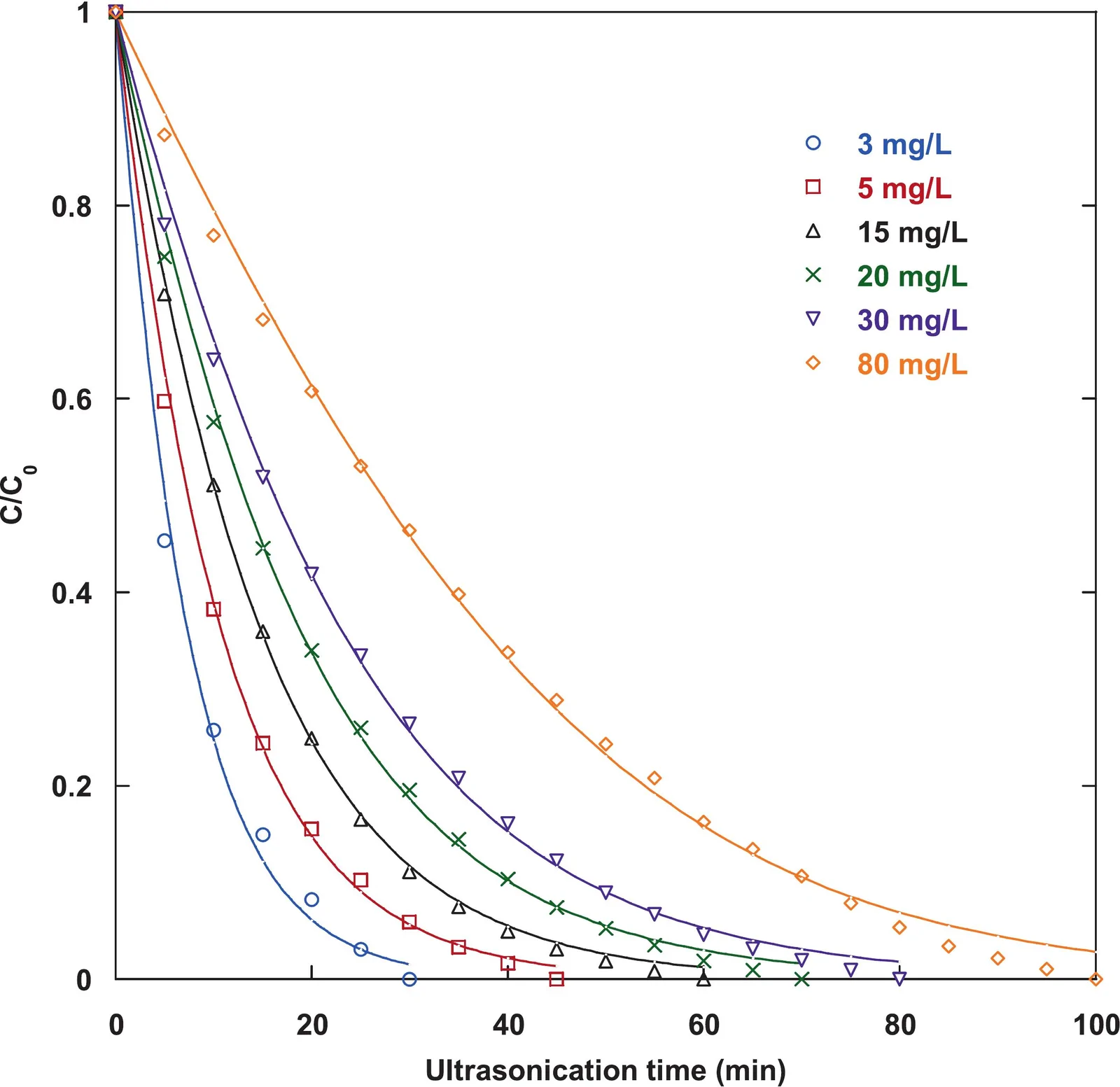
Experimental and globally fitted normalized concentration profiles (C/C_0_) for NBB sonodegradation at initial concentrations of 3, 5, 15, 20, 30, and 80 mg/L. Symbols represent experimental observations, and solid lines represent four-parameter Langmuir–Freundlich calibration fits. Internal out-of-sample performance was evaluated separately by blocked leave-one-concentration-out validation.

Fig. 3 presents the experimental NBB profiles together with the corresponding global four-parameter calibration fits. The individual-profile calibration RMSE values ranged from 0.0134 to 0.0332 in normalized concentration units. These values characterize in-sample representation only; transfer to observations excluded from parameter estimation is evaluated separately below.

The time-resolved exponent, n_T_ = 0.9422, was higher than the initial-rate exponent, n = 0.8256, although both values remained below unity. The two analyses describe related but distinct aspects of the concentration response. The initial-rate model quantifies how the early degradation rate varies with initial pollutant loading, whereas the time-resolved formulation describes the complete depletion trajectories as the instantaneous concentration decreases during irradiation. The approximate 95 % confidence interval of n_T_, 0.8902–0.9942, approaches but does not exceed the classical Langmuir limit of unity, indicating a mildly sub-Langmuir dynamic response within the investigated range.

The concentration dependence of the normalized profiles also demonstrates the limitation of a concentration-independent pseudo-first-order interpretation. If NBB degradation were governed by a single first-order coefficient, the normalized C/C_0_ profiles would exhibit comparable decay patterns across the investigated concentration range. Instead, normalized depletion became progressively slower as C_0_ increased. This systematic loading dependence is represented by the coupled Langmuir–Freundlich occupation term and the continuous loading-dependent capacity function.

Internal out-of-sample transfer across pollutant loading was assessed using blocked leave-one-concentration-out validation. In each fold, one complete concentration–time profile was withheld, the four common parameters were estimated from the remaining profiles, and the excluded trajectory was calculated without parameter re-estimation. The pooled cross-validated RMSE was 0.0391. The individual validation R^2^ values ranged from 0.9577 to 0.9969, and the corresponding RMSE values ranged from 0.0164 to 0.0614. The largest error occurred when the 80 mg/L profile, located at the upper boundary of the complete-profile concentration range, was withheld. These values quantify internal transfer to an unobserved concentration profile under otherwise identical experimental conditions.

A separate concentration-endpoint assessment was performed using the 30-min NBB removal measurements. Complete concentration–time profiles at 3, 5, 15, 20, 30, and 80 mg/L were used for calibration, whereas the measurements at 7, 10, 40, and 120 mg/L were not used to estimate any time-resolved parameter. At these four withheld concentrations, the calculated 30-min removal efficiencies were 94.23 %, 91.18 %, 70.62 %, and 44.66 %, compared with experimental values of 91.16 %, 89.81 %, 70.32 %, and 43.46 %, respectively. The resulting RMSE was 1.79 percentage points; the corresponding R^2^ = 0.9914 is reported descriptively because only four held-out concentration endpoints were available.

The formulation therefore permits interpolation of concentration trajectories and treatment performance across pollutant loading without fitting a separate capacity coefficient at every concentration. Its parameters remain specific to NBB, the investigated sonoreactor, acoustic conditions, temperature, gas atmosphere, and solution matrix. The validation performed here does not establish transfer beyond that experimental domain.

### 4.4 Time-resolved modeling and loading-based validation of furosemide degradation

**Fig. 4:**
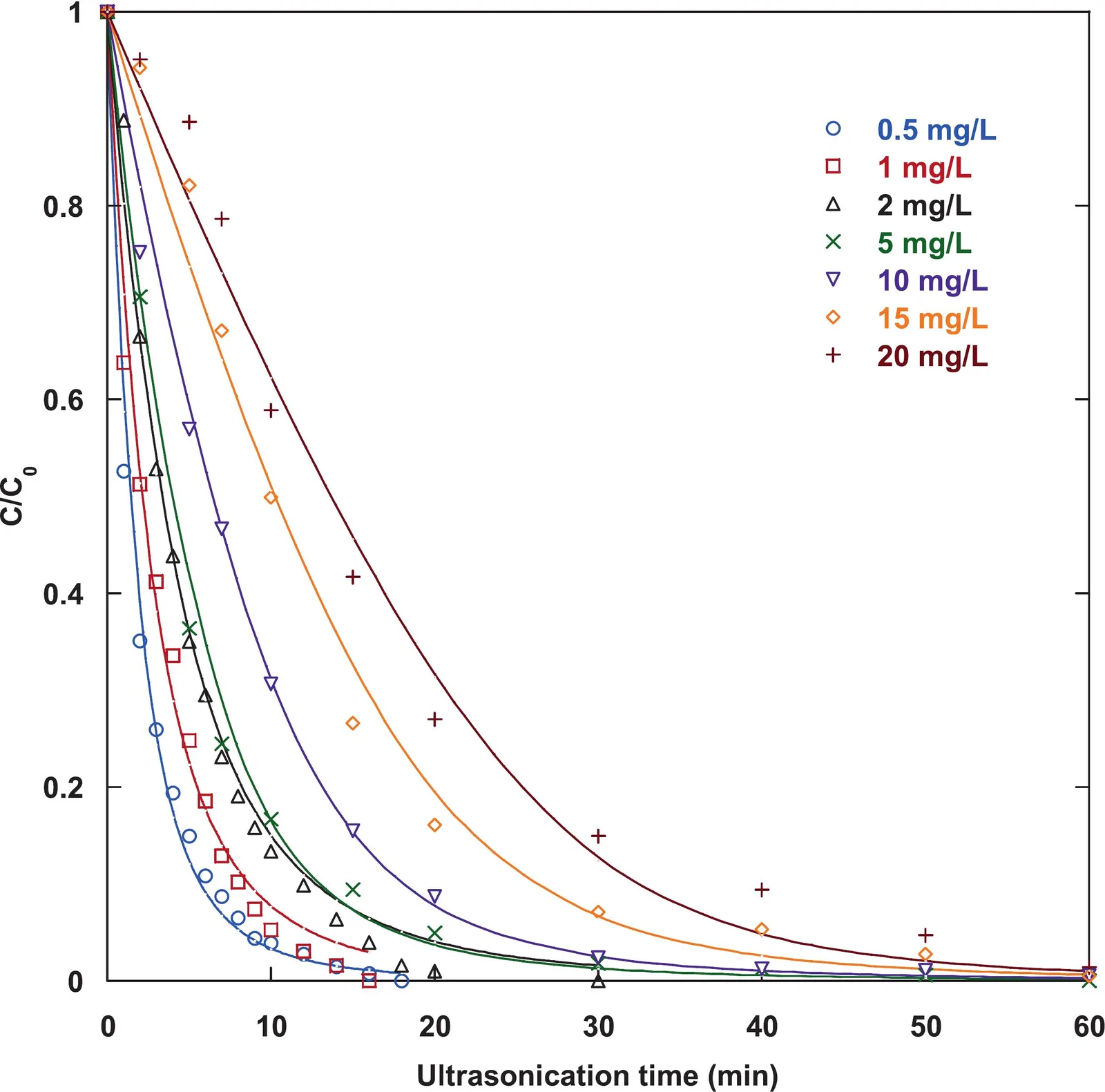
Experimental and globally fitted normalized concentration profiles (C/C_0_) for furosemide sonodegradation at initial concentrations of 0.5, 1, 2, 5, 10, 15, and 20 mg/L. Symbols represent experimental observations, and solid lines represent four-parameter Langmuir–Freundlich calibration fits. Internal out-of-sample performance was evaluated separately by blocked leave-one-concentration-out validation.

The time-resolved Langmuir–Freundlich formulation was applied to the normalized degradation profiles of furosemide over the initial concentration range of 0.5–20 mg/L (Fig. 4). All concentration–time profiles were fitted simultaneously using the four common parameters r_0,ref,T_, β_T_, K_LF,T_, and n_T_. No concentration-specific capacity coefficient was estimated.

Using Cref = 10 mg/L, global nonlinear regression gave: r_0,ref,T_ = 0.9224 ± 0.0377 mg/L·min, β_T_ = 0.5467 ± 0.0604, K_LF,T_ = 0.1023 ± 0.0191 L/mg, and n_T_ = 1.2378 ± 0.0536.

The corresponding apparent half-response concentration was:

(37) $$C_{1/2,T}=\frac{1}{K_{LF,T}}=9.77\;mg/L$$

The loading-dependent apparent oxidative-capacity function was:

(38) $$R_{T}\left(C_{0}\right)=1.8190{\left(\frac{C_{0}}{10}\right)}^{-0.5467}\;mg/L\cdot min$$

The complete numerical rate expression for furosemide was therefore:

(39) $$-\frac{dC}{dt}=1.8190{\left(\frac{C_{0}}{10}\right)}^{-0.5467}\frac{{\left(0.1023\;C\right)}^{1.2378}}{1+{\left(0.1023\;C\right)}^{1.2378}}$$

The global calibration RMSE was 0.0399 in normalized concentration units. Parameter estimates and uncertainty are summarized in Table 2. Relative standard errors ranged from 4.09 % to 18.62 %, and the pairwise parameter-correlation structure is reported in Table 4.

**Table 2:** Global time-resolved parameter estimates and uncertainty for furosemide degradation.

**Parameter or statistic**	**Value**	**RSE (%)**	**Approximate 95 % CI**
r_0,ref,T_ (mg/L·min)	0.9224 ± 0.0377	4.09	0.8485–0.9964
β_T_	0.5467 ± 0.0604	11.04	0.4285–0.6650
K_LF,T_ (L/mg)	0.1023 ± 0.0191	18.62	0.0650–0.1397
n_T_	1.2378 ± 0.0536	4.33	1.1328–1.3428
C_1/2,T_ (mg/L), calculated	9.77	−	7.16–15.39

Note: All concentration–time profiles were fitted simultaneously using four common parameters and C_ref_ = 10 mg/L. Values following ± are standard errors obtained from nonlinear regression. C_1/2,T_ = 1/K_LF,T_ is a calculated quantity and was not independently fitted.

Fig. 4 presents the experimental furosemide profiles together with the corresponding global four-parameter calibration fits. The formulation represented the transition from rapid normalized depletion at low initial concentration to slower depletion at higher loading without assigning an independently adjustable coefficient to each profile. This visual and statistical agreement concerns observations used in parameter estimation and therefore represents calibration performance only. Internal out-of-sample performance is evaluated separately by the blocked leave-one-concentration-out procedure.

Internal out-of-sample transfer across pollutant loading was evaluated by blocked leave-one-concentration-out validation. In each fold, one complete concentration–time profile was withheld, the four parameters were estimated from the remaining profiles, and the excluded trajectory was calculated without parameter re-estimation. The pooled cross-validated RMSE was 0.0566. The largest errors occurred when profiles near the lower boundary of the investigated concentration domain were withheld. Because no additional furosemide dataset collected at independent concentrations or under independent operating conditions was available, these results are interpreted as internal cross-validation rather than external experimental validation.

The fitted value n_T_ = 1.2378 describes the curvature of the furosemide concentration dependence within the analyzed dataset. Because n_T_ was obtained by nonlinear regression rather than measured independently, it should not be interpreted as a direct adsorption-cooperativity or interfacial-heterogeneity coefficient. In the Langmuir–Freundlich expression, n_T_ > 1 produces a superlinear concentration dependence in the low-occupation region followed by saturation at higher concentration; it does not imply that degradation accelerates as irradiation proceeds. The value may incorporate the combined effects of contaminant speciation, interfacial access, radical-interception efficiency, mass transfer, intermediate formation, and the transient cavitation environment. It is therefore treated as an apparent dynamic descriptor rather than evidence of positive molecular cooperativity at the bubble–liquid interface.

The four-parameter formulation provides a continuous relationship between initial pollutant loading and the complete concentration trajectory. Its parameters remain specific to furosemide and to the applied reactor, acoustic intensity, temperature, gas atmosphere, and solution matrix. Quantitative application beyond this domain requires independent recalibration and validation.

### 4.5 Compound-dependent apparent kinetic behavior

The apparent parameters obtained for furosemide and NBB differed substantially within their respective experimental domains. Both contaminants are effectively nonvolatile under the investigated conditions, making direct transfer into the cavitation-bubble gas phase unlikely. Nevertheless, the present macroscopic kinetic data do not permit the parameter differences to be assigned uniquely to hydrophobicity, adsorption, interfacial enrichment, radical interception, or any other individual molecular property [[5], [7], [8], [9], [15], [16], [22]].

Furosemide gave K_LF,T_ = 0.1023 L/mg, whereas NBB gave K_LF,T_ = 5.322 × 10^−3^ L/mg. The corresponding apparent half-response concentrations were 9.77 and 187.91 mg/L, respectively. These values indicate that the fitted furosemide response changed curvature over a lower bulk-concentration range than the fitted NBB response. They should not be interpreted as independently measured adsorption constants or direct interfacial coverages.

The different concentration windows used for the two contaminants also reflect their markedly different aqueous solubilities. The higher solubility of NBB permitted investigation over a substantially broader mass-concentration range, whereas the lower solubility of furosemide necessitated a lower concentration domain to preserve dissolved-phase conditions throughout the kinetic experiments. These compound-specific windows were therefore selected to obtain meaningful loading-response information within experimentally accessible concentration domains rather than to enable a direct comparison at identical concentrations. The markedly different fitted half-response concentrations further illustrate why identical concentration ranges in mg/L would not necessarily sample equivalent regions of the respective concentration–response curves.

The fitted dynamic exponents were n_T_ = 1.2378 for furosemide and n_T_ = 0.9422 for NBB. The NBB confidence interval approached the classical Langmuir limit of unity, whereas the furosemide interval remained above unity. These exponents quantify the curvature of the respective concentration responses but do not establish adsorption heterogeneity or molecular cooperativity.

The loading-response exponent was larger for furosemide, with β_T_ = 0.5467, than for NBB, with β_T_ = 0.3354, indicating a stronger decrease in the fitted apparent capacity scale with increasing furosemide loading. However, the compounds were investigated within different concentration domains, reflecting their distinct aqueous solubilities, and at different acoustic intensities. Consequently, the fitted parameters represent combined compound–reactor–condition responses and should not be interpreted as intrinsic molecular constants or used for direct quantitative ranking of the two contaminants.

The comparison therefore establishes compound-dependent differences in the apparent concentration scale, loading dependence, and curvature of the macroscopic kinetic response. It does not establish the microscopic origin of those differences. Independent experiments controlling acoustic conditions and directly probing interfacial accumulation or radical reaction pathways would be required for that purpose.

### 4.6 Comparison with simpler models, parameter identifiability, and practical applicability

Table 3 compares the calibration and internal leave-one-concentration-out performance of the loading-dependent pseudo-first-order, classical Langmuir, and Langmuir–Freundlich formulations. For NBB, the Langmuir–Freundlich formulation produced the smallest calibration residual but the largest cross-validated error, whereas the loading-dependent pseudo-first-order formulation gave the lowest error for the withheld profiles. Thus, the additional Langmuir–Freundlich exponent is not supported by predictive performance alone for NBB.

**Table 3:** Comparison of the time-resolved kinetic formulations.

**Compound**	**Formulation**	**Number of parameters**	**Calibration RMSE**	**Pooled internal leave-one-concentration-out RMSE**
NBB	Loading-dependent pseudo-first-order	2	0.0249	0.0281
NBB	Classical Langmuir (n_T_ = 1)	3	0.0205	0.0329
NBB	Langmuir–Freundlich	4	0.0196	0.0391
Furosemide	Loading-dependent pseudo-first-order	2	0.0522	0.0605
Furosemide	Classical Langmuir (n_T_ = 1)	3	0.0445	0.0558
Furosemide	Langmuir–Freundlich	4	0.0399	0.0566

Note: RMSE values are expressed in normalized concentration units. Calibration RMSE was calculated from observations used for parameter estimation. For blocked leave-one-concentration-out validation, one complete concentration–time profile was withheld in each fold and calculated without parameter re-estimation. Lower calibration error does not necessarily indicate lower out-of-sample error; model selection should therefore consider parameter number and cross-validated performance together.

For furosemide, the Langmuir–Freundlich formulation again produced the lowest calibration error, whereas its cross-validated performance was essentially equivalent to, and slightly poorer than, that of the classical Langmuir model. The additional exponent therefore captures curvature within the fitted dataset but does not provide a decisive out-of-sample advantage.

These results demonstrate why model selection cannot be based solely on calibration goodness of fit. The additional Langmuir–Freundlich exponent should be retained only when its uncertainty, parameter correlations, residual behavior, and out-of-sample performance collectively justify the additional degree of freedom. Under the present conditions, the loading-dependent pseudo-first-order formulation is the most parsimonious predictive option for NBB, whereas the classical Langmuir and Langmuir–Freundlich formulations show comparable cross-validated performance for furosemide.

As defined in Section 3.4, the diagnostic modules and the time-resolved predictor are estimated separately. The core time-resolved formulation therefore requires four shared parameters rather than one concentration-specific coefficient per kinetic profile. Table 3 further shows that, when justified by out-of-sample performance, the practical predictive model can be reduced to the two-parameter loading-dependent pseudo-first-order or three-parameter classical Langmuir formulation.

The correlation matrices in Table 4 reveal strong compensation among the initial-rate parameters, particularly between r_max_ and K_LF_, whereas the four-parameter time-resolved formulations show lower but still non-negligible correlations. These results indicate that the measured concentration responses constrain combinations of parameters more strongly than they constrain each physical contribution independently. The covariance analysis therefore supports local practical identifiability of the time-resolved fits but does not establish global structural identifiability.

**Table 4:** Pairwise correlation matrices for the fitted kinetic parameters.

**(a) NBB initial-rate parameters**				
**Parameter**	**r_max_**	**K_LF_**	**n**	
**r_max_**	1	−0.996	−0.944	
**K_LF_**	−0.996	1	0.960	
n	−0.944	0.960	1	
**(b) NBB time-resolved parameters**				
**Parameter**	r_0,ref,T_	β_T_	K_LF,T_	n_T_
r_0,ref,T_	1	0.810	0.369	0.833
β_T_	0.810	1	−0.071	0.717
K_LF,T_	0.369	−0.071	1	0.592
n_T_	0.833	0.717	0.592	1
**(c) Furosemide time-resolved parameters**				
**Parameter**	r_0,ref,T_	β_T_	K_LF,T_	n_T_
r_0,ref,T_	1	0.722	−0.467	0.556
β_T_	0.722	1	−0.392	0.766
K_LF,T_	−0.467	−0.392	1	0.242
n_T_	0.556	0.766	0.242	1

Note: Correlations were calculated from the covariance matrix at the nonlinear-regression optimum. These values describe local parameter compensation and do not constitute a global structural-identifiability analysis.

The parameters have restricted physical meanings. The reference-rate parameter establishes the degradation-rate scale at C_ref_; β_T_ quantifies the empirical loading dependence of the apparent oxidative capacity; K_LF,T_ defines an apparent concentration scale; and n_T_ describes curvature. None is a direct measurement of surface coverage, adsorption free energy, interfacial area, radical flux, or molecular cooperativity. The fitted value n_T_ > 1 for furosemide is therefore treated as an apparent shape parameter and not as evidence of positive cooperative accumulation at the cavitation interface.

A practical application should proceed by collecting concentration–time profiles at multiple pollutant loadings spanning the intended operating range, fitting the candidate loading-dependent pseudo-first-order, classical Langmuir, and Langmuir–Freundlich formulations, and selecting the simplest model supported by blocked out-of-sample performance. The selected formulation may then be used to interpolate C(t), removal efficiency, and the irradiation time required to reach a specified residual concentration under the same experimental conditions. This workflow is calibration-based and is not an a priori molecular-property predictor. Recalibration and independently collected validation data are required when the contaminant, reactor geometry, acoustic frequency, delivered power, gas atmosphere, temperature, or solution matrix changes.

The contribution of the framework therefore lies not in universal predictive superiority or proof of a unique interfacial mechanism, but in providing a modular and physically motivated representation that connects loading-dependent kinetics, apparent saturation behavior, net H_2_O_2_ response, parameter uncertainty, and explicit model comparison. The simpler alternatives define the circumstances under which the additional Langmuir–Freundlich exponent is or is not supported.

## 5 Conclusions

A parsimonious loading-response framework was evaluated for the sonochemical degradation of the nonvolatile contaminants NBB and furosemide. Ultrasound-off controls showed that the concentrations of both contaminants remained essentially unchanged under stirring-only conditions, confirming that non-sonochemical pollutant losses were negligible under the investigated conditions. Concentration-specific time-resolved capacity coefficients were replaced by a continuous loading-dependent function, allowing each complete kinetic dataset to be represented using four globally estimated parameters. Parameter uncertainty and covariance analysis showed that the initial-rate formulation exhibited substantial parameter compensation, whereas the reference-rate time-resolved parameterization provided improved practical identifiability.

Calibration performance was clearly distinguished from out-of-sample assessment. Internal blocked leave-one-concentration-out validation evaluated transfer to withheld concentration profiles, while additional 30-min NBB measurements at 7, 10, 40, and 120 mg/L provided a held-out concentration-endpoint assessment not used for time-resolved parameter estimation. These tests support interpolation across pollutant loading under fixed experimental conditions but do not establish external transfer to another reactor, acoustic condition, water matrix, or independently collected furosemide dataset.

Comparison with simpler alternatives showed that the Langmuir–Freundlich formulation was not universally superior in prediction. The loading-dependent pseudo-first-order model gave the lowest cross-validated error for NBB, whereas the classical Langmuir and Langmuir–Freundlich formulations showed essentially comparable predictive performance for furosemide. The additional Langmuir–Freundlich exponent should therefore be retained only when its uncertainty and out-of-sample performance justify the added flexibility.

The value n_T_ > 1 obtained for furosemide is interpreted as an apparent curvature parameter rather than evidence of positive molecular cooperativity. Similarly, the inverse association between NBB degradation and net H_2_O_2_ formation is treated as an empirical concentration–response relationship and not as a complete radical-partition balance. The fitted parameters consequently represent compound-, reactor-, and condition-specific descriptors rather than universal molecular constants.

Within its calibrated domain, the selected formulation can be used to interpolate concentration trajectories, calculate removal efficiency, and estimate the irradiation time required to attain a specified treatment objective. Application to another contaminant or operating environment requires recalibration and independently collected validation data.

## CRediT authorship contribution statement

**Oualid Hamdaoui:** Writing – review & editing, Writing – original draft, Visualization, Validation, Supervision, Software, Resources, Project administration, Methodology, Investigation, Funding acquisition, Formal analysis, Data curation, Conceptualization.

## Declaration of competing interest

The author declares that they have no known competing financial interests or personal relationships that could have appeared to influence the work reported in this paper.

## References

[1] Serna-Galvis E.A., Porras J., Torres-Palma R.A. (2022). A critical review on the sonochemical degradation of organic pollutants in urine, seawater, and mineral water. Ultrason. Sonochem..

[2] Richardson S.D., Kimura S.Y. (2020). Water analysis: Emerging contaminants and current issues. Anal. Chem..

[3] Suslick K.S. (1990). Sonochemistry. Science.

[4] Thompson L.H., Doraiswamy L.K. (1999). Sonochemistry : Science and Engineering. Ind. Eng. Chem. Res..

[5] Hoffmann M.R., Hua I., Höchemer R. (1996). Application of ultrasonic irradiation for the degradation of chemical contaminants in water. Ultrason. Sonochem..

[6] Hu D., Liu S., Zhang G. (2025). Sonochemical treatment for removal of aqueous organic pollutants: principles, overview and prospects. Sep. Purif. Technol..

[7] Pétrier C., Francony A. (1997). Ultrasonic waste-water treatment: Incidence of ultrasonic frequency on the rate of phenol and carbon tetrachloride degradation. Ultrason. Sonochem..

[8] Okitsu K., Iwasaki K., Yobiko Y., Bandow H., Nishimura R., Maeda Y. (2005). Sonochemical degradation of azo dyes in aqueous solution: A new heterogeneous kinetics model taking into account the local concentration of OH radicals and azo dyes. Ultrason. Sonochem..

[9] Nanzai B., Okitsu K., Takenaka N., Bandow H. (2009). Sonochemical degradation of alkylbenzene sulfonates and kinetics analysis with a Langmuir-type mechanism. J. Phys. Chem. C.

[10] Alrashed M., Hamdaoui O. (2024). True order determination of sonochemical degradation kinetics of organic contaminants in water using the half-lives method. J. Eng. Res..

[11] Adewuyi Y.G. (2001). Sonochemistry : Environmental science and engineering applications. Ind. Eng. Chem. Res..

[12] Kidak R., Ince N.H. (2006). Ultrasonic destruction of phenol and substituted phenols: A review of current research. Ultrason. Sonochem..

[13] Serpone N., Terzian R., Hidaka H., Pelizzetti E. (1994). Ultrasonic induced dehalogenation and oxidation of 2-, 3-, and 4-chlorophenol in air-equilibrated aqueous media. Similarities with irradiated semiconductor particulates. J. Phys. Chem..

[14] Kidak R., Ince N.H. (2008). A novel adsorption/saturation approach to ultrasonic degradation of phenol. J. Adv. Oxid. Technol..

[15] Chiha M., Merouani S., Hamdaoui O., Baup S., Gondrexon N., Pétrier C. (2010). Modeling of ultrasonic degradation of non-volatile organic compounds by Langmuir-type kinetics. Ultrason. Sonochem..

[16] Hamdaoui O. (2023). General analytical solution expressions for analyzing Langmuir-type kinetics of sonochemical degradation of nonvolatile organic contaminants in water. Ultrason. Sonochem..

[17] Sips R. (1948). On the structure of a catalyst surface. J. Chem. Phys..

[18] Ferkous H., Hamdaoui O., Merouani S. (2015). Sonochemical degradation of naphthol blue black in water: Effect of operating parameters. Ultrason. Sonochem..

[19] Gasmi I., Hamdaoui O., Ferkous H., Alghyamah A.A. (2023). Sonochemical advanced oxidation process for the degradation of furosemide in water: Effects of sonication’s conditions and scavengers. Ultrason. Sonochem..

[20] Merouani S., Hamdaoui O., Saoudi F., Chiha M. (2010). Influence of experimental parameters on sonochemistry dosimetries: KI oxidation, Fricke reaction and H2O2 production. J. Hazard. Mater..

[21] Kormann C., Bahnemann D.W., Hoffmann M.R. (1988). Photocatalytic production of H_2_O_2_ and organic peroxides in aqueous suspensions of TiO_2_, ZnO, and desert sand. Environ. Sci. Technol..

[22] Lu W., Dong D., Xu L., Zhang Y., Han J., Mei X., Qiao W., Shen X., Gan L. (2024). Sulfite activation by ultrasound for the degradation of emerging contaminants: The dependence of pollutant property and mechanistic study. Sep. Purif. Technol..

